# No stone unturned: Prevalence effects in interactive search are different than those in visual search

**DOI:** 10.3758/s13423-026-02919-2

**Published:** 2026-04-29

**Authors:** Haden Dewis, Cheryl D. Metcalf, Martin B. Warner, Richard Polfreman, Hayward J. Godwin

**Affiliations:** 1 School of Psychology, University of Southampton, Highfield, Southampton, Hampshire SO17 1BJ UK; 2 School of Healthcare Enterprise & Innovation, Faculty of Medicine, University of Southampton, Southampton, UK; 3 School of Health Sciences, University of Southampton, Southampton, UK; 4 Department of Music, University of Southampton, Southampton, UK

**Keywords:** Interactive search, Low prevalence, Visual search

## Abstract

When carrying out a search for a target object, manipulation with the environment may be required to successfully detect the target. These searches are known as *interactive searches*. Many real-life examples of interactive search involve searching for targets that are unlikely to be present or are said to have low target prevalence. To date, the effects of low target prevalence upon interactive search behaviors remain unclear. We conducted two experiments to examine search exhaustiveness in interactive search, focusing on whether searchers were less exhaustive when prevalence was low, primarily in terms of behavior during target-absent trials. For both experiments, we found a standard effect of low prevalence on response accuracy, such that low-prevalence targets were more likely to be missed than high-prevalence targets. However, through the utilization of Bayesian analyses, we found strong evidence against the influence of prevalence upon response times and all other search exhaustiveness measures during target-absent trials. In other words, contrary to traditional visual search findings, changes in response accuracy were not a result of reductions in search exhaustiveness. We conclude that, during interactive search, even when prevalence is low, searchers operate under a no-stone-unturned approach. Under this approach, searchers are unwilling to provide an “absent” response without checking most—if not all—possible places, regions or areas in a display that could contain a target.

During an interactive search, an observer must physically interact with objects or change their own viewing position to either find a target or confirm its absence (Hout et al., 2022; Sauter et al., 2020). Unlike static laboratory-based visual search tasks, in everyday life, interactive search is the norm rather than the exception. For example, a simple search within our bags for our house keys, the cupboard for a snack, or our desks for a pen. Interactive search is also required for the detection of critical targets in a range of security scenarios such as airport baggage screening and forensic searches (Dewis et al., 2025; Godwin et al., 2024; Riggs et al., 2017, 2018). A common issue in these applied search settings is the role of target prevalence. Target prevalence refers to the proportion of searches wherein a target appears (Horowitz, 2017; Wolfe et al., 2007). It is well established within visual search that when prevalence is low, response times (RTs) decrease and response accuracy declines (Wolfe et al., 2005, 2007); this is known as the prevalence effect. Here, we examine how the prevalence effect manifests itself within interactive search, with a specific focus on whether shifts in prevalence influence how exhaustive searchers are.

It is important to explore exhaustiveness during low-prevalence interactive search because within visual search tasks, when target prevalence is low, exhaustiveness has been shown to reduce. This reduction in exhaustiveness manifests as a decrease in the number of objects examined (Godwin, Menneer, Cave, et al., 2015a, 2015b; Rich et al., 2008) and a reduction in the likelihood of fixating target objects (Godwin, Menneer, Cave, et al., 2015a, 2015b). Likewise, in the rare cases where low-prevalence targets are successfully fixated, they are often incorrectly rejected as being distractors (Godwin, Menneer, Riggs, et al., 2015a, 2015b; Hout et al., 2015). Whilst the prevalence effect is well understood within visual search, no past research has examined the prevalence effect within interactive search tasks. Studying the prevalence effect in interactive search can therefore help to address a key theoretical issue and difference between visual and interactive search that has not yet been examined to date. During visual search, when a response is made, all available visual information has been presented to the searcher (even if it has not been directly fixated or examined). This is not necessarily the case in an interactive search, as often at least some visual information is obscured until it is revealed by the searcher. The primary goal of our experiments here was therefore to examine the effects of low prevalence on interactive search. We did this primarily be examining exhaustiveness in low-prevalence interactive search. Put another way, we sought to determine whether participants would leave “no stone unturned,” even if they expected each proverbial stone to be unlikely to have a target upon it.

The only theoretical model that accounts for interactive search is that of Hout et al. (2022; see Fig. 1). Whilst this model does capture search termination, it does not include reference to how comprehensively or exhaustively individuals are willing to search prior to termination. Given the previously mentioned research on prevalence and eye movements (e.g., Godwin, Menneer, Cave, et al., 2015a, 2015b; Godwin, Menneer, Riggs, et al., 2015a, 2015b; Hout et al., 2015; Rich et al., 2008; Wolfe et al., 2007), it seems unlikely that searchers will remain exhaustive in their interactive searching when targets are rare. Likewise, research into foraging suggests that time and energetic resources should be directed towards areas that provide the most benefit to the searcher (e.g., Bremset Hansen et al., 2009; Fryxell, 1991; Van Beest et al., 2010). As such, exhaustively checking for targets that are rarely present is not an optimal approach and seems unlikely (Ehinger & Wolfe, 2016). In addition to simply not checking objects, it has also been shown that searchers reduce the time they spend examining objects when prevalence is low (Peltier & Becker, 2016). In other words, when the target is rare, the speed at which searchers inspect objects increases. As such, it seems probable that in a low-prevalence interactive search, searchers will do similar by increasing the speed at which they manipulate and interact with objects as they become more willing to forgo careful inspection. We then have two routes by which targets can easily be missed during interactive search: first, by never revealing them, and second by examining them so briefly (or even not at all) that they are missed (e.g., Godwin, Menneer, Riggs, et al., 2015a, 2015b; Hout et al., 2015).

**Fig. 1:**
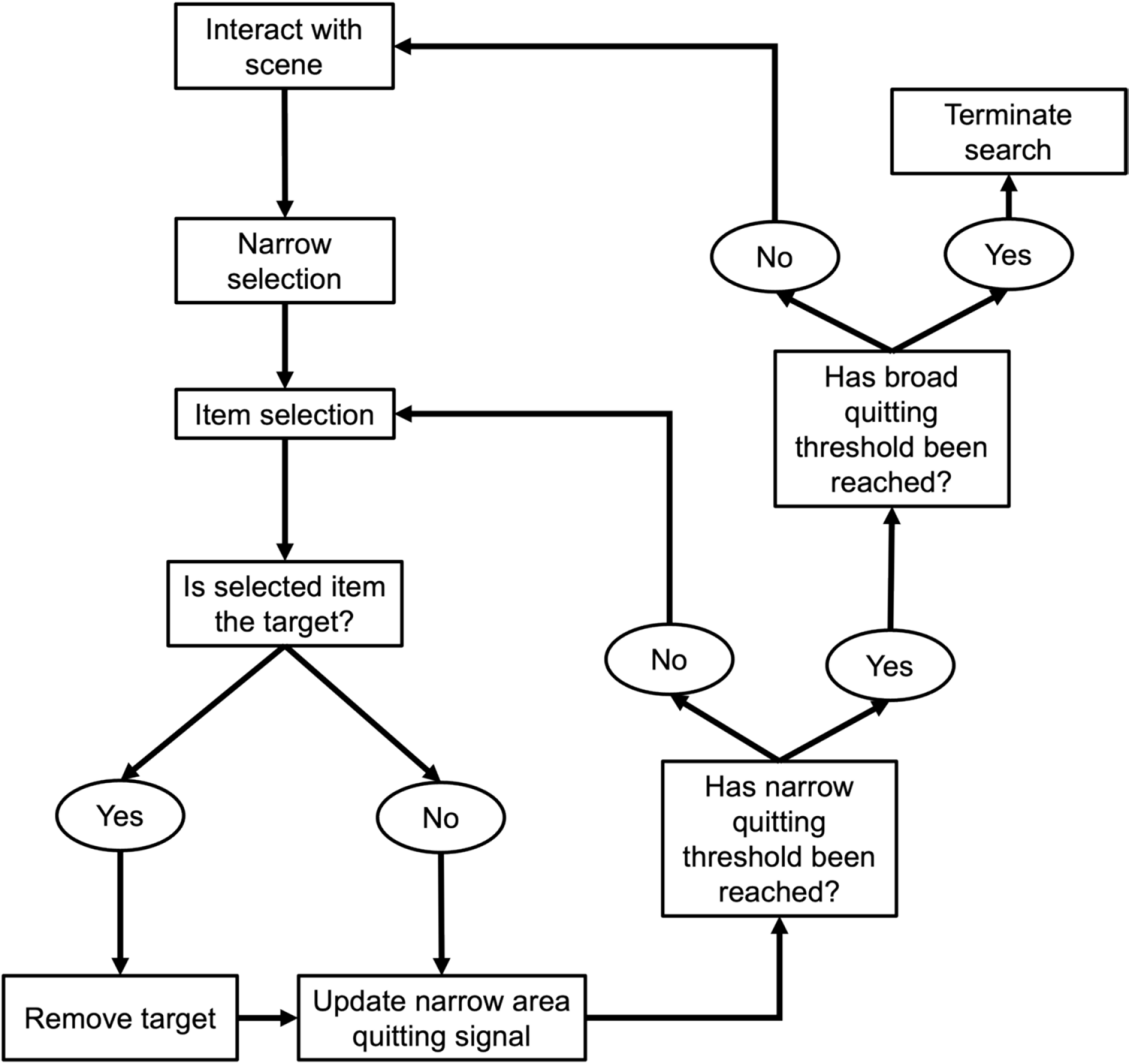
Interactive multiple decision model (Hout et al., 2022). *Note*. Hout et al.’s (2022) interactive multiple decision model (i-MDM). According to the i-MDM, the visual field is broken down into narrow areas that have a high likelihood of containing the target. The area with the highest density of target-relevant features will be attended to first. An item is selected, and a two-alternative force choice is made regarding whether the item is a target or a distractor. If the selected item is not the target, then another decision must be made regarding continuing the search in the specified narrow area. If the selected item is the target, then the item is removed, and search continues in the narrow area until the quitting threshold is met. Once this threshold is met, the searcher may then choose to (or choose not to) interact with the scene before selecting the next narrow area to investigate

Here, we report the results of two experiments that build upon past work in interactive search (Dewis et al., 2025). We asked participants to search for a *T* shape placed upon the side of a set of virtual cubes that participants could rotate and examine (see Fig. 2). In both experiments, we manipulated target prevalence. In Experiment 1, the number of virtual cubes per trial was limited to four, however, in Experiment 2, we increased the number of cubes a participant needed to search through from four cubes per trial to eight. Our goal within Experiment 2 was to further understand how much visual information participants were willing to leave unchecked when the requirements for an exhaustive search increase, whether this would be further influenced by low prevalence, and again its influence upon the speed of interactions.

**Fig. 2:**
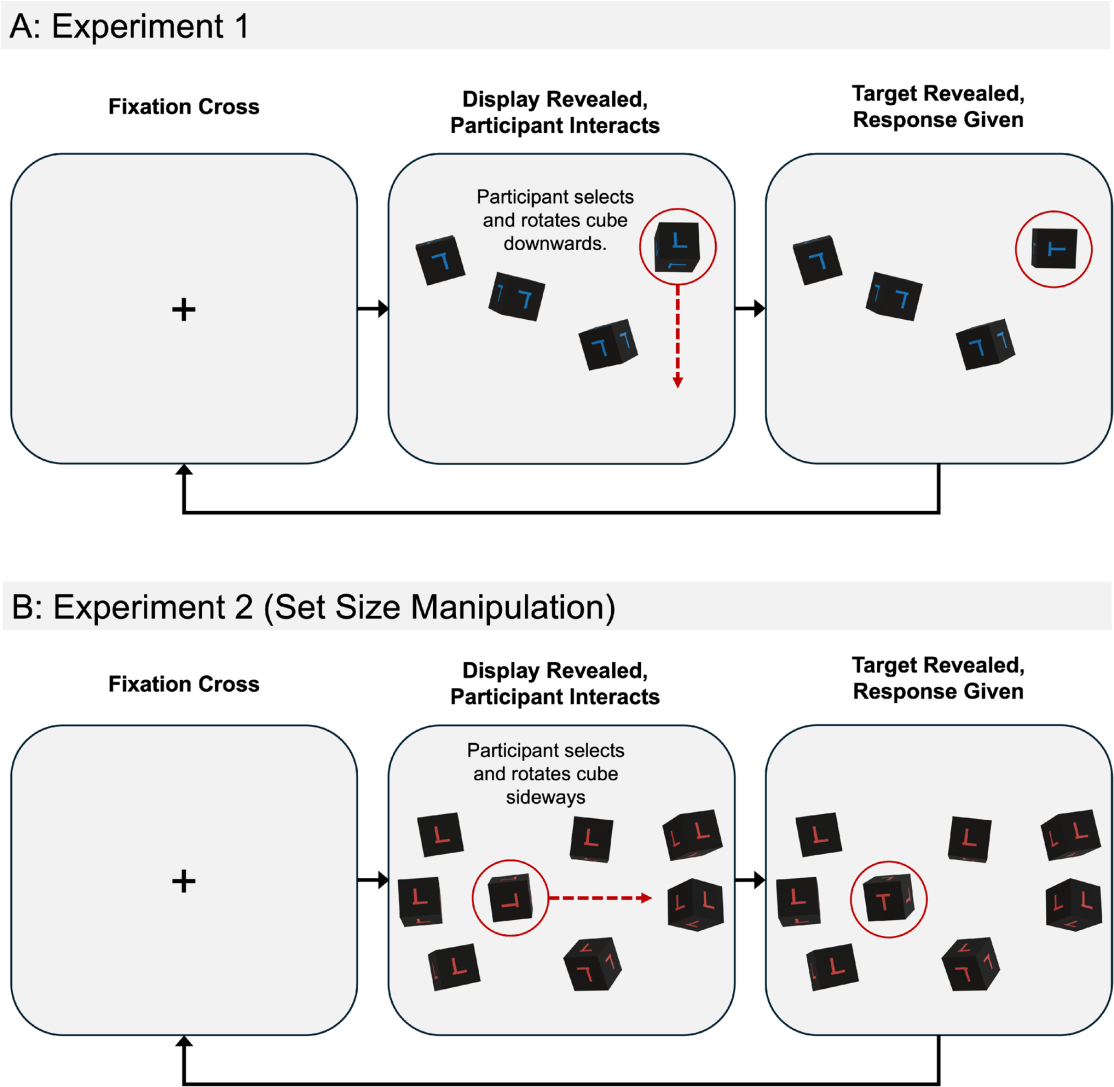
Trial procedure for each experiment. *Note*. Figure depicts the procedure of a typical trial for both Experiment 1 and Experiment 2. The red circles and arrows were not visible to the participant and are included here to aid visibility. Upon reveal of the display, participants interacted with and rotated cubes using their cursor once the target was found (or deemed absent), the participant ended the trial with a keyboard press. This whole process then repeated for 120 trials. (Color figure online)

We predicted the following: (1) Response accuracy would decrease when target prevalence was low; (2) reductions in target-absent RTs would occur when prevalence was low due to early search termination; (3) participants would become less exhaustive in their searching of target-absent trials as they learnt that the target was rarely present—as evidenced by the proportion of visual information a participant reveals; (4) when target prevalence was low, the speed at which participants checked through cubes would increase as they would not expect objects to contain the target.

## Methods

### Ethical approval

Ethical approval was given by the University of Southampton’s Ethics Committee on the October 20, 2023 (ERGO NUMBER: 89065) for Experiment 1 and on July 11, 2024 (ERGO NUMBER: 89065.A5) for Experiment 2.

### Open practices statement

We report how we determined our sample size, data exclusions, all manipulations, and all measures in the study. Data, materials, and analysis code for all experiments in this study can be accessed online via this web address (https://osf.io/v954y). Experiments were not preregistered.

### Participants

A priori power analyses were carried out for both experiments using pilot data from 20 participants, using the *simr* package in R (Green & MacLeod, 2016). Power analyses were conducted for each dependent variable being analyzed. Target effect sizes were based on prior research (Dewis et al., 2025; Godwin et al., 2024) to avoid the issues associated with “observed power” (see Hoenig & Heisey, 2001, for an explanation). These analyses confirmed that a minimum sample size of 40 participants was required to achieve a power level of 0.80 for Experiment 1 and 50 participants for Experiment 2.

For Experiment 1, 54 participants were recruited from the University of Southampton (*M*_age_ = 19.18 years, *SD* = 1.14, women = 82.35%, men = 11.76%, nonbinary = 2.94%, rather not say = 2.94%) during December 2023 and January 2024 and received course credits for their participation.

For Experiment 2, 50 new participants were recruited from the online participant recruitment platform Prolific (*M*_age_ = 36.86, *SD* = 12.44, women = 44.00%, men = 56.00%) during July 2024. Participants were paid £12.00 for taking part. The Prolific platform allows researchers to set several filters to restrict participation to certain sets of individuals. In Experiment 2, we utilized these tools to apply the following filters when advertising our study: 1) Only include individuals who report themselves as fluent English speakers from the UK; 2) only include individuals with a Prolific approval rating of 95% or above (i.e., in 95% of the studies they participated in, researchers deemed their data sets as acceptable, with no flaws or failures of attention tests); 3) only include individuals who have reported having normal or corrected-to-normal vision; 4) only include participants who report having normal color vision. Our reason for doing so was to ensure the highest possible level of data quality.

### Stimuli and apparatus

Stimuli were created using the open-source software Blender (Hess, 2010). Interactive displays were rendered using a Three.js (an open-source JavaScript library that allows three-dimensional graphics to be displayed and interacted with within a web browser) and jsPsych (an open-source JavaScript library used for building web-based psychological experiments) framework (Danchilla, 2012; De Leeuw, 2015).

Stimuli consisted of different cubes with *L* and *T* shapes placed onto their faces. Cubes consisted of either a single distractor* L* shape on each of their six faces, or a single distractor *L* shape on five of their six faces and a target *T* shape on the remaining sixth face. The color of these *T* and *L* shapes varied between participants and was selected at random from a set of 16 colors that have been used in previous visual search studies (Godwin et al., 2016; Godwin, Menneer, Riggs, et al., 2015a, 2015b; Stroud et al., 2012). For each trial, cubes were randomly placed into the search array using a 5 × 3 grid and then randomly rotated through all axes around their point of origin by up to 360º. For Experiment 1, four cubes per trials were used, and for Experiment 2 this was increased to eight cubes per trial.

Participants completed the study using their own computers or laptops and associated peripherals. They were told to press the *M* key of their keyboard if they believed the search array contained a target shape and the *Z* key of their keyboard if they believed the search array did not contain a target shape. Cubes were interacted with by selecting a cube and dragging their cursor across the screen; the cube would then rotate in the direction of the cursor movement.

### Design and procedure

After consenting to take part, participants were provided with detailed instructions regarding the task followed by a training segment that allowed them to practice rotating a cube for as long as they needed. Participants then went on to complete five practice trials with feedback regarding accuracy, before starting the real trials. All participants then completed a total of 120 trials over the course of ~45 min. On each trial, a fixation cross was displayed for 500 ms before the search array was revealed. The search array remained on-screen until a response was given. Following a participant response, the search array was removed, the trial ended, and the next trial’s fixation cross displayed. Following the practice trials, participants did not receive trial-by-trial feedback regarding accuracy. A typical target-present trial is depicted in Fig. 2A for Experiment 1 and Fig. 2B for Experiment 2.

Participants were randomly allocated to the low-prevalence group (target shape present on 10% of trials) or the high-prevalence group (target shape present on 50% of trials) at the start of the experiment. All remaining trials were target-absent trials.

## Results

### Data cleaning

Prior to all analyses, data underwent preplanned cleaning. As little is known about interactive search behaviors, and these experiments are exploratory by nature, very little data were removed during this process. We opted to remove data based solely upon RTs across three steps. First, participants with average by-trial RTs longer than 60 s (120 s for Experiment 2) were removed. During piloting, we found that on average, exhaustive search took ~20 s (~40 s for Experiment 2) per target-absent trial. As such, we chose an average RT cutoff that was three times this value (as is typically done with standard deviation; e.g., Eskenazi, 2023; Godwin et al., 2021; Osborne, 2010). To obtain such a large average RT, participants would have had a substantial number of extremely long search trials. Should this have been the case, this would likely have been a result of the participant not engaging in the task properly and therefore needed to be removed. Second, we removed any trials longer than 180 s (360 for Experiment 2). We again chose this value as it is three times larger than our average cutoff value. Finally, we removed any trials shorter than 250 ms. Within our data analyses, if a stimulus was visible within the display for at least 250 ms, then we concluded this visual information to have been visible to the searcher; we have detailed our reasons for doing this within the relevant section below. As such, a small number of trials shorter than 250 ms could not be analyzed and were therefore removed. Following all cleaning steps, the final datasets consisted of 6,290 trials from 53 participants (24 from low prevalence, 29 from high prevalence) for Experiment 1 and 5,977 trials from 50 participants (24 from low prevalence, 26 from high prevalence) for Experiment 2. A breakdown of the steps taken and the removed data can be found in Table 1.

**Table 1:** Data-cleaning steps for each experiment

	Experiment 1 (4 cubes)		Experiment 2 (8 cubes)	
Removal step	Trials removed	Remaining trials	Trials removed	Remaining trials
Raw data	0 (0.00 %)	6,480 (100.00 %)	0 (0.00 %)	5,997 (100.00 %)
Exceeded average cut-off	120 (1.85 %)	6,360 (98.15 %)	0 (0.00 %)	5,997 (100.00 %)
Long trials	13 (0.20 %)	6,347 (97.95 %)	10 (0.17 %)	5,987 (99.83 %)
Short trials	57 (0.87 %)	6,290 (97.07 %)	10 (0.17 %)	5,977 (99.67 %)

Exceeded average cut-off = participants’ average RTs > 60 s for Experiment 1 and > 120 s for Experiment 2; long trials = individual RTs > 180 s for Experiment 1 and > 360 s for Experiment 2; short trials = individual RTs < 250 ms for both experiments.

### Analytic approach

Effects were modelled through Bayesian linear and generalized linear mixed-effects models (BLMM, BGLMM) within R (R Core Team, 2023) via the *brms* package (Bürkner, 2017) and findings confirmed using Bayes factors calculated via the *bayestestR* package (Makowski et al., 2019). Bayes factors are the result of a likelihood ratio test between an alternative hypothesis and a null hypothesis. Bayes factors greater than 1.00 indicate stronger evidence towards the alternative hypothesis, whilst Bayes factors of less than 1.00 suggest stronger evidence towards the null hypothesis. For an effect to be deemed trustworthy, it required both a 95% credible interval (CI) that did not pass through zero, and a Bayes factor of greater than 3. We employed a Bernoulli distribution with a logit link for modelling binary measures, a Gaussian distribution with log-transformed dependent variables for modelling RT measures, and an inflated Beta distribution for modelling proportional measures. Models used the same following fixed factors where relevant: presence (target-absent, target-present), prevalence (10%, 50%), and object order (a continuous measure of order of object interactions). The random effects structure for all models contained random intercepts for participants and random intercepts and slopes for target initial visibility (a measure of whether the target was immediately visible to the participant within a trial).

We analyzed standard response accuracy and RT measures in addition to several measures inspired by those found within eye-tracking research (see Godwin et al., 2021, for examples). These included the proportion of visual information revealed, and the speed at which participants rotated objects. To compute the proportion of visual information revealed, we measured the number of non-visible cube faces a participant revealed across trials and converted this value into a proportion. A cube face was counted as being “revealed” when it was visible within the display for longer than 250 ms. This value was chosen as it is often used as a fixations are typically ~250 ms in duration and thus are often used as a minimum time requirement for registering successful object identification within eye tracking research (Godwin et al., 2021; Liversedge et al., 2011; Rayner & Pollatsek, 2006). Additionally, this value has been successfully used within previous interactive search experiments (Dewis et al., 2025). Whilst we acknowledge that identification can happen at a faster rate (e.g., Kirchner & Thorpe, 2006), we have erred on the side of caution here, potentially underestimating effects, to ensure that we have only counted a face as being revealed if it truly was.

### Findings

**Fig. 3:**
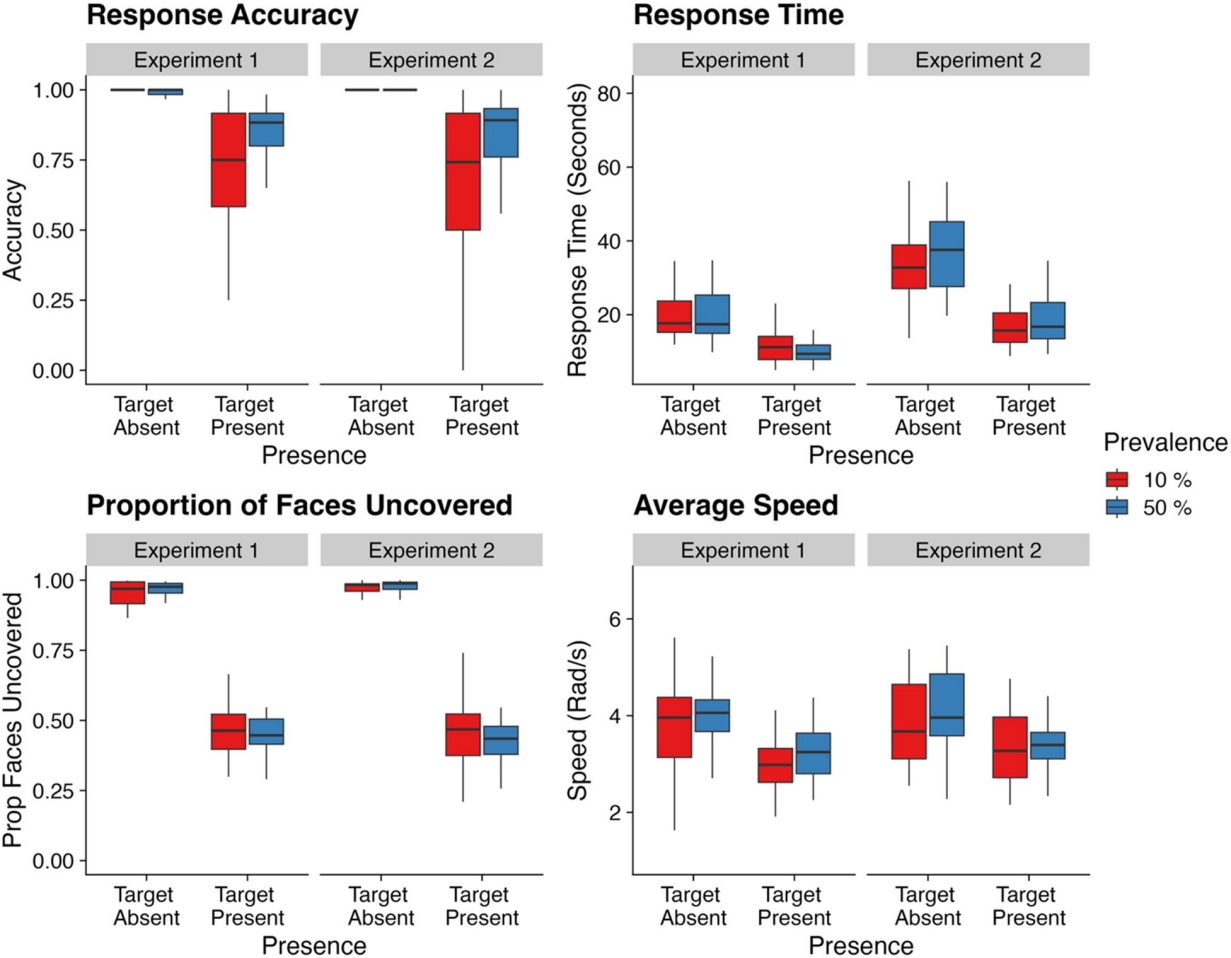
Response accuracy rates, response times, proportion of faces uncovered as a function of presence, prevalence, and experiment. *Note*. Box plots display descriptive statistics for each analysis within the current studies. Whiskers indicate the maximum and minimum values of each group. (Color figure online)

We report effects from all analyses within Tables 2 and 3, and visually depicted descriptives in Fig. 3. As can be seen within the top left panel of Fig. 3 we observed a standard low-prevalence effect upon response accuracy. However, as can be seen within the remaining panels, to our surprise, the effect of prevalence appeared to be nonexistent across our other measures. We will now better examine these apparent null results using Bayesian statistics.

**Table 2:** Modelled effects and Bayes factors for Experiment 1

	Accuracy			RT		
Parameter	Estimate	CI	BF_10_	Estimate	CI	BF_10_
Intercept	3.36 (0.19)	**3.00, 3.74**	**7.00×10**^**20**^	9.16 (0.08)	**9.00, 9.32**	**7.99×10**^**129**^
Prevalence (low, high)	0.00 (0.34)	−0.67, 0.65	0.33	−0.12 (0.10)	−0.32, 0.08	0.21
Presence (absent, present)	−3.69 (0.21)	**−4.11, −3.30**	**7.32×10**^**21**^	−0.58 (0.03)	−**0.65,** −**0.52**	**2.50×10**^**19**^
Prevalence × presence	1.46 (0.39)	**0.71, 2.22**	**218.19**	−0.06 (0.06)	−0.19, 0.05	0.11
	Visual information revealed			Speed		
Parameter	Estimate	CI	BF_10_	Estimate	CI	BF_10_
Intercept	0.49 (0.05)	0.40, 0.58	**2.28×10**^**10**^	3.34 (0.17)	**3.02, 3.67**	**7.59×10**^**19**^
Prevalence (low, high)	−0.04 (0.08)	−0.21, 0.12	0.10	0.15 (0.20)	−0.25, 0.54	0.26
Presence (absent, present)	−1.06 (0.06)	**−1.17, −0.94**	**6.14×10**^**16**^	−0.24 (0.06)	**−0.35, −0.13**	**218.84**
Prevalence **×** presence	−0.07 (0.12)	−0.30, 0.16	0.14	0.08 (0.11)	−0.14, 0.30	0.14

CIs = credible intervals; BF = Bayes factor; boldfaced CI values = CIs that did not pass through zero; boldfaced BF values = BF > 3.20. Values in parentheses represent the associated standard error values. All *R*-hat values = 1.00. Effects were deemed reliable if CIs did not pass through zero and BF > 3.00.

**Table 3:** Modelled effects and Bayes factors for Experiment 2

	Accuracy			RT		
Parameter	Estimate	CI	BF_10_	Estimate	CI	BF_10_
Intercept	3.36 (0.19)	**2.99, 3.75**	**1.24×10**^**20**^	9.90 (0.07)	**9.76, 10.05**	**7.53×10**^**149**^
Prevalence (low, high)	0.49 (0.35)	−0.20, 1.18	0.92	0.08 (0.11)	−0.13, 0.29	0.14
Presence (absent, present)	−3.66 (0.20)	**−4.07, −3.28**	**4.47×10**^**22**^	−0.73 (0.03)	**−0.79, −0.67**	**4.96×10**^**28**^
Prevalence **×** presence	0.81 (0.37)	**0.06, 1.54**	**3.75**	0.00 (0.06)	−0.11, 0.11	0.06
	Visual Information Revealed			Speed		
	Estimate	CI	BF_10_	Estimate	CI	BF_10_
Intercept	0.77 (0.03)	**0.71, 0.84**	**1.29×10**^**28**^	3.6 (0.17)	**3.26, 3.93**	**7.44×10**^**21**^
Prevalence (low, high)	0.03 (0.06)	−0.08, 0.15	0.07	0.01 (0.27)	−0.52, 0.54	0.27
Presence (absent, present)	−1.69 (0.06)	**−1.80, −1.58**	**5.87×10**^**35**^	−0.25 (0.05)	**−0.34, −0.16**	**3,270**
Prevalence **×** presence	0.14 (0.11)	−0.07, 0.34	0.25	0.12 (0.09)	−0.07, 0.30	0.20

CIs = credible intervals; BF = Bayes factor; boldfaced CI values = CIs that did not pass through zero; boldfaced BF values = BF > 3.20. Values in parentheses represent the associated standard error values. All *R*-hat values = 1.00. Effects were deemed reliable if CIs did not pass through zero and BF > 3.00.

As stated above, we found the standard effects of prevalence upon response accuracy rates. This was evident across both experiments within a presence × prevalence interaction (Experiment 1, high-prevalence present vs. low-prevalence present: Estimate = −0.74, lower CI = −1.38, upper CI = −0.07, BF_10_ = 3.27. Experiment 2, high-prevalence present vs. low-prevalence present: Estimate = −0.89, lower CI = −1.55, upper CI = −0.22, BF_10_ = 9.65).

Despite this, prevalence had little influence anywhere else. In RTs, we failed to find the standard effects of low prevalence on target-absent trials: in fact, we found strong evidence *against* any changes in RTs for target-absent trials for both experiments (BF_10_ values ≤ 0.11).

With regard to search exhaustiveness, to our surprise, we again found strong evidence *against* any effects of prevalence (BF_10_ values ≤ 0.25), participants remained extremely exhaustive within target-absent trials, revealing on average ~95% of visual information across both experiments.

Likewise, within our analyses of speed, we again found strong evidence *against* any effects of prevalence (BF_10_ values ≤ 0.27). Despite objects rarely containing the target within the low-prevalence condition, the average speed at which participants rotated and manipulated cubes remained consistent between prevalence conditions, averaging at ~4 rad/s for both prevalence conditions over both experiments.

However, across all analyses we found that when comparing target-present trials to target-absent trials, RTs were quicker, the quantity of visual information uncovered was less, and the average speed of rotations was slower. These effects are best explained as a result of early trial termination following target detection. In these cases, the participant would not need to interact with all four cubes thus resulting in quicker RTs, less visual information being revealed, and more variable speed data.

Overall, then, participants were exhaustive in their searches and typically refused to terminate a target-absent trial before having revealed all faces of each cube. Unlike visual search, target prevalence did not influence participants’ willingness to conduct an interactive search exhaustively, nor did it influence the speed at which they interacted with objects.

## Discussion

Our goal here was to examine exhaustiveness in interactive search, focusing on whether searchers are less exhaustive when prevalence is low, primarily in terms of behavior during target-absent trials. We did so across two experiments in which participants completed a varied prevalence (10%, 50%) interactive search for a *T* shape attached to the side of a set of virtual cubes that could be rotated. In Experiment 2, we increased the number of cubes to search through from four per trial to eight with the goal of taxing the limits of participants’ willingness to search exhaustively by increasing the effort required for an exhaustive search.

For both experiments, we found a standard effect of low prevalence on response accuracy, such that low-prevalence targets were more likely to be missed than high-prevalence targets. This alone replicates the basic findings of visual search experiments. However, to our surprise, we found no evidence in either experiment that RTs were reduced during target-absent trials when prevalence was low, a finding that runs counter to one of the main and highly replicated results in the visual search low-prevalence literature (Horowitz, 2017; Rich et al., 2008; Wolfe et al., 2005, 2007). At a basic level this suggests that the effects of low prevalence in interactive search are fundamentally different to those in visual search. We have reached this conclusion since we have found evidence of a prevalence effect upon accuracy but not RTs, which are typically used to examine search exhaustiveness.

More in-depth analyses conducted on both experiments further confirmed this surprising finding: Participants were equally exhaustive in terms of the proportion of cube faces revealed in high and low prevalence across both experiments; participants did not move the cubes faster under the expectation that no target would be present in either experiment when prevalence was low.

This leads to two interesting questions: (1) why do we still observe a prevalence effect despite participants not differing in terms of their exhaustiveness? (2) What is so different about interactive search that searchers are unwilling to terminate searches before having revealed all visual information even when the visual information they reveal so rarely contains the target?

With regard to the first question, as with static visual search, a lack of search exhaustiveness is not the only factor contributing towards the low-prevalence effect. It is often suggested that reductions in response accuracy when target prevalence is low are a result of a criterion shift (e.g., Wolfe et al., 2007; Wolfe & Van Wert, 2010). In other words, searchers become more conservative in their willingness to accept an object as the target (due to the rare rate at which the target appears) and therefore become more likely to incorrectly reject the target as a distractor when it does finally appear. We believe that this is likely also happening here. However, our low-prevalence effect was not quite as strong as what is typically found within the visual search literature. This may be due to a lack of feedback given to participants (see Lyu et al., 2021, for an overview) or could simply be a fundamental difference between interactive and visual search altogether. Although participants in the low-prevalence condition were overall less accurate than those in the high-prevalence condition, accuracy for target-present trials across both conditions was also not particularly high^1^—in a standard visual search for *T* shapes, accuracy is usually at or near ceiling (see University of Southampton Psychology Collaboration et al., 2026, for an example). Remarkably, across both experiments, 95.87% (*SD* = 27.80) of incorrect target-present trials for those in the high-prevalence condition and 91.62% (*SD* = 19.92) for those in the low-prevalence condition occurred when participants had either revealed the target or the target was plainly visible. In other words, despite interacting with target objects, participants still frequently failed to identify them. Whilst surprising, this finding is not new and has been reliably observed across numerous sets of experiments conducted by Solman et al. (2012) using their virtual interactive unpacking paradigm. Solman and colleagues put this phenomenon down to a reduced cognitive ability to juggle both physical interactions and visual processing simultaneously (e.g., Goodale & Milner, 1992; Jeannerod, 1994); something that fundamentally sets visual and interactive search apart. Indeed, this difference may further explain the weaker prevalence effect observed here.

Returning to question 2, why, despite the search arrays rarely containing the target in the low-prevalence condition, did participants remain exhaustive in their searching? One possible reason for this is that our set size was not large enough to find evidence of a shift in exhaustiveness. Despite the fact that Experiment 1 presented only four cubes per trial, the true set size of objects to be searched (the *T*s and *L*s attached to the sides of the four cubes) was 24 items per trial, similar to that of a normal visual search task (e.g., Wolfe, 2020). Moreover, we doubled the number of cubes that participants needed to search through in Experiment 2 and still found a near identical pattern of results. In fact, to better look into this issue, we compared differences within search exhaustiveness between the two experiments to better confirm the influence of changes in the number of cubes to search through (see Table 4). As with all other analyses, we again found no evidence of the influence of prevalence upon search exhaustiveness as measured by the proportion of faces revealed (BF_10_ = 0.10), or an interaction between prevalence and the number of cubes to search through (BF_10_ = 0.33). In fact, we found that compared to Experiment 1, those within Experiment 2 revealed an overall greater absolute proportion of faces (~3% more) than those within Experiment 1. If anything, then, the increase in cubes within Experiment 2 made participants more exhaustive, albeit to a very small extent, and made no difference to the prevalence effect.

**Table 4:** Modelled effects and Bayes factors for visual information revealed ~ prevalence and experiment

Parameter	Estimate	CIs	BF_10_
Intercept	1.67 (0.05)	**1.58, 1.76**	**1.02×10**^**40**^
Prevalence (low, high)	0.02 (0.09)	−0.16, 0.21	0.10
Experiment (one, two)	0.73 (0.09)	**0.55, 0.92**	**1.75×10**^**7**^
Prevalence × experiment	−0.20 (0.19)	−0.58, 0.17	0.33

CIs = credible intervals; BF = Bayes factor; boldfaced CI values = CIs that did not pass through zero; boldfaced BF values = BF > 3.20. Values in parentheses represent the associated standard error values. All *R*-hat values = 1.00. Effects were deemed reliable if CIs did not pass through zero and BF > 3.00.

These findings are similar to the debate surrounding satisfaction of search (SoS; Berbaum et al., 1990, 2000, 2015). In SoS, searchers were said to become “satisfied” following the detection of a target and consequently terminate their searches early/search less exhaustively. However, Berbaum et al. (1991) showed this not to be the case. In their study, radiologists were provided with views of different sets of chest X-rays and asked to locate any abnormal nodules. Within this task, it was found that regardless of target detection, searchers searched exhaustively for similar durations. As such, they argued that SoS effects were *not* a result of early trial termination but instead that available perceptual resources diminished over time resulting in missed targets. This phenomenon has since become known as “subsequent search misses” (see Adamo et al., 2021, for a review) and research has converged on the idea that it is not related to early quitting but instead that initial target detection consumes working memory resources that would otherwise aid in finding subsequent targets (Adamo et al., 2021; Cain et al., 2014; Cain & Mitroff, 2013). Indeed, it seems that we have observed something similar within our task. Instead of terminating searches early, searchers may have become interested in reaching an arbitrary threshold such as interacting with all stimuli or searching for a set period of time regardless of the likelihood that the array contained the target.

The high levels of search exhaustiveness we observed may also be closely linked to metacognitive ability (see Norman et al., 2019, for a review). When conducting a static visual search, it is easier to *think* that all areas within the display have been carefully checked, even when they have not been. As such, termination may therefore occur before all items have been inspected (e.g., Godwin, Menneer, Riggs, et al., 2015a, 2015b; Hout et al., 2015). In our interactive search, however, it may be harder to know what one has and has not checked—the visual system must keep track of six faces per cube that appear, disappear, and reappear as the cube is rotated through three-dimensional space. As such, searchers may become more careful in their searching in an attempt to reassure themselves that they have checked all faces of all the cubes; this may indeed be why we observed a small increase in exhaustiveness in Experiment 2. Of course, the opposite may also be true, and searchers may in fact be capable of keeping perfect track of which objects and faces they have and have not inspected. Although we increased the number of cubes in Experiment 2, we still did not find the limit to participants’ search exhaustiveness. In a messier, more realistic real-world search, it may be harder to keep track of these things, and it is likely that searchers would become less exhaustive. All this to say, further research is warranted and necessary to further understands the limits of the effects observed throughout this study.

Overall, we conclude that, during interactive search, even when prevalence is low, searchers operate under a “no stone unturned” approach. Under this approach, it seems to be the case that searchers are unwilling to provide an “absent” response without checking most—if not all—possible places, regions or areas in a display that could contain a target. This is particularly interesting because it demonstrates that even well-established findings in visual search do not replicate or translate to interactive search. Moreover, it demonstrates that searchers are unwilling to terminate a search based on partial or incomplete information (i.e., they need to experience some sense of checking the entire environment including hidden areas).

Textbooks or introductory chapters often begin by providing examples of visual search that are in fact interactive. For example, in Chun and Wolfe (1996), the authors state within the second sentence of their manuscript: “You are searching for that piece of paper among a mess of various articles, journals, forms, and other miscellaneous paperwork on your desk.” This is undoubtedly a task that typically requires interaction. Likewise, Eckstein (2011) suggests that searching for “one's vehicle in a parking lot” or “keys in a living room” are both examples of visual search. However, when carefully considering these tasks, both require body movements and interactions to locate these targets. The authors cited here are of course in no way intentionally deceiving readers, but it does highlight the need for careful consideration of the differences between interactive and visual search. Perhaps, as we have shown here, further careful study and experimentation will demonstrate that visual search is in fact not a good approximation of interactive search after all.

## Data Availability

We report how we determined our sample size, data exclusions, all manipulations, and all measures in the study. Data, materials, and analysis code for all experiments in this study can be accessed online via this web address: https://osf.io/v954y Experiments were not preregistered.

## Footnotes

1 The accuracy statistics for target-present trials across all experiments are as follows: Experiment 1, low-prevalence target-present trials: *M* = 72.14, *SD* = 44.91. Experiment 1, high-prevalence target-present trials: *M* = 85.77, *SD* = 34.94. Experiment 2, low-prevalence target-present trials: *M* = 68.88, *SD* = 35.94. Experiment 2, high-prevalence target-present trials: *M* = 84.77, *SD* = 46.38.
